# Medical and Philosophical News

**Published:** 1783

**Authors:** 


### SECTION III.

### Medical and Philosophical News.

THE Royal Medical Society at Paris have proposed the following subject for a prize of 60 livres value: "To determine what diseases, whether acute or chronic, are to be considered as truly contagious; in what manner each of these diseases may be communicated by one individual to another; and what are the most certain means of stopping the progress of these different contagions." Dissertations on this subject, in French or Latin, will be received by M. Vicq D'Azyr, secretary to the society, till the 1st of January, 1785.

The Royal Academy of ſurgery at Paris have propoſed the following ſubject for a prize medal of the value of 500 livres: "Determiner les "differentes conſtitutions des ſtylets ou ſondes "ſolides et des ſondes cannelées; quels ſont les "cas où elles doivent etre admiſes, ſuivant leur "forme particuliere, et quelle eſt la methode "d'en faire uſage." The Diſſertations on this ſubject are to be written in French or Latin, and ſent to M. Louis, ſecretary of the academy, before December 31, 1783.

---

The Harveian Society of Edinburgh have propoſed the following ſubject for a prize medal: "An inquiry concerning the nature and pro-"perties of the Peruvian Bark, and the compa-"rative powers of the red and quilled Peruvian "Bark." The Diſſertations muſt be written in Latin or Engliſh, and delivered to doctors Duncan or Webſter, ſecretaries to the ſociety, on or before the 1ſt of January 1784.

---

In

In the *Journal de Medecine* for Auguſt 1783, M. Brillouet, ſurgeon at Paris, has added another inſtance to the few already upon record, of the complication of the Small Pox with the Meaſles.——On the 6th of April 1783, he inoculated the two ſons of the Viſcount de Virie, and another child. Two of the patients had a ſlight eruption of ſmall pox on the 10th day after inoculation, and went through the diſeaſe without experiencing any uncommon ſymptom. The viſcount's eldeſt ſon, a child five years old, began to ſicken at the ſame time as the other two, viz. on the 8th day. On the 10th his fever was abated, he was lively, and the progreſs of the local ſymptoms ſeemed to be ſtopped. The next day the fever returned with increaſed violence, and attended with ſpaſms. On the 12th three variolous puſtules were diſcovered, but without any abatement of the ſymptoms. He was now much troubled with cough, and his eyes appeared inflamed and watery. On the 13th he had a complete eruption of the meaſles. The alarming ſymptoms now diminiſhed, and in three days the meaſles diſappeared. During this time the ſmall pox ſeemed to be at a ſtand, but the day following, viz. on the 16th after inoculation, he had a freſh acceſs of fever, ſucceeded ceeded by a very confluent ſmall pox, from which he with difficulty recovered.

In the ſame work M. Trabuc, ſtudent of ſurgery at Aix, has related the caſe of a patient whoſe death was occaſioned by an abſceſs in the left iliac region, which on diſſection was found to have its origin in the kidney of that ſide. The kidney was of an extraordinary ſize, and in its tubular ſubſtance were found ſixteen calculi of different ſizes. Each of the calculi was lodged in a diſtinct cell, formed by a very thin cellular membrane. The reſt of the kidney was in a ſtate of ſuppuration.

---

M. Semin, ſurgeon to the Hotel Dieu at Narbonne, has lately publiſhed in the *Gazette de Santé*, a curious inſtance of the effects of maſturbation in a ſhepherd, named Gabriel Galien, who began to addict himſelf to this baneful practice at the age of fifteen, and at firſt repeated it ſeven or eight times a day.——After a while the pleaſure he experienced from this manœuvre began to diminiſh, and when he was about ſix and twenty years old, the repetition of it ceaſed to excite any ſenſation. At this time he diſcovered, vered, that by irritating the end of the urethra with a little wooden ftick, he could ftill fatisfy himfelf completely. This artifice, however, in procefs of time failed to produce the defired effect, the urethra being by degrees rendered infenfible, and even callous.——Lamenting this lofs of fenfibility, he now determined to try the effect of a flight incifion at the end of the urethra. This he did with the point of his knife, and inftead of pain, it produced, as he afterwards affured Mr. Semin, an agreeable fenfation, and the effect he wifhed for.

Pleafed with the fuccefs of this experiment, he often repeated it, and by flitting open from time to time a little of the urethra, in the courfe of two years, the glans penis, urethra, and corpora cavernofa were completely divided up to the pubis. When he happened, as he did fometimes, to bring on an hæmorrhage, he ftopped it by tying a piece of packthread round his penis.

As he could now no longer ufe his knife, he was obliged to have recourfe again to the ufe of a little ftick, and with this he was able to reach the orifice of the veficulæ feminales, and bring on an emiffion of femen. He continued to repeat this practice till June 1774, when when he one day plunged the ſtick ſo far into the remaining part of the urethra, that it eſcaped from his fingers and paſſed into the bladder. Soon after this he was brought to the Hotel Dieu at Narbonne, and there in the preſence of the two phyſicians of the hoſpital, and of eight ſurgeons of that city, Mr. Semin, extracted the extraneous ſubſtance from his bladder by the lateral operation. The patient, who had been ſubject to cough and purulent expectoration for ſome time before, died fifty-eight days after the operation, at the age of thirty-ſix years.

On diſſection the lungs were found adhering every where to the pleura, and in their right lobe was diſcovered a large abſceſs. The pericardium adhered to the heart, which was flaccid, with its coronary veſſels in a varicous ſtate.—— The urinary bladder was ſound, with a firm cicatrix; the reſt of the abdominal viſcera abounded with adheſions, but afforded no other particular marks of diſeaſe.

———

The following information relative to the natural hiſtory of the Red Peruvian Bark is extracted from a letter addreſſed by Dr. Simmons to to Dr. Saunders, and inferted by the latter in the third edition of his work on that fubject:

" Amongft the papers of the late M. Jofeph " de Juffieu, (brother of the famous Bernard de " Juffieu) one of the French academicians, who " went to Quito in Spanifh America, in order " to afcertain the figure of the earth, and who " died lately at Paris, feveral interefting obfer- " vations have been found relative to the Pe- " ruvian Bark. Thefe have been communicated " to the Royal Medical Society at Paris, by his " nephew Dr. Anthony de Juffieu. In his de- " fcription of the genus, M. de Juffieu agrees " with his fellow traveller, M. de la Condamine, " but he admits a greater number of fpecies. " Thefe, however, may perhaps very properly " be reduced to two, as the reft feem to be " only varieties.

" The firft fpecies includes the red, the yel- " low, and the knotty (*le noueux*) Barks, all of " which have very fmooth leaves, flowers of a " purplifh colour, and inodorous, with a bark " that is bitter to the tafte, and more or lefs co- " loured. Of thefe three the Red is held in " the higheft eftimation, and it is this fort of " Bark, according to M. de Juffieu, which was " employed in the early days of this remedy in " Europe, and which acquired it fo much, and

" ſuch deſerved celebrity. The tree that pro-

" duces it is become ſo exceeding ſcarce, that

" in the year 1739, M. de Juſſieu found it

" growing only in a few places in the neigh-

" bourhood of Loxa, ſo that the inhabitants of

" Peru had been obliged to ſubſtitute the yel-

" low and knotty Barks in its ſtead, both of

" which they are ſaid to prefer for their own

" uſe, becauſe they ſuppoſe them to be leſs ac-

" tive and heating. But M. de Juſſieu, who

" had experienced the good effects of the Red

" Bark, both in his own perſon, and in others,

" conſidered it as infinitely ſuperior to the reſt.

" Even the trees that produce the yellow and

" knotty Barks, are ſaid to be diminiſhing in

" number ſo faſt, that it is to be feared they

" will in time become extinct, unleſs a regular

" mode of cultivating them is adopted, or they

" are diſcovered elſewhere.

" The ſecond ſpecies includes the White Barks,

" of which there are four varieties. They have

" all of them broad roundiſh hairy leaves; the

" flowers are red, very odoriferous, and fur-

" niſhed with hairs on their inſide ſurface. The

" fruit is longer than that of the former ſpecies,

" and the outer bark is of a whitiſh colour. In

" two of theſe varieties, the inner layers of the

" bark

" bark are of a reddiſh hue; they have a ſlightly " bitter taſte, and when freſh are ſaid to poſſeſs " a ſlight febrifuge quality, but which they ſoon " loſe. The bark of the other two is intirely " white, inſipid, and of no efficacy.

" M. Ant. de Juſſieu has ſtill in his poſſeſſion " ſome extract prepared by his uncle upwards " of forty years ago at Loxa, from the Red " Bark. Some trials lately made with it, prove " it to be infinitely ſuperior in efficacy to the ex- " tract of Bark in common uſe, ſo that its vir- " tues do not ſeem to have been diminiſhed by " keeping.

" M. de Juſſieu, in his travels, found a few " of the trees that produce the yellow and knot- " ty Barks, growing in different parts of the " valley that extends along the chain of the " Andes, and in the diſtrict of Yungas, which " is near it; but it was only about Loxa, in the " 4th degree of S. Lat. that he ſaw the foreſts " of thoſe trees. It would ſeem therefore, that " the heat peculiar to ſuch a latitude is more " genial to the *Cinchona* than that of any other " climate, and of courſe we can hope to meet " with it only in ſuch a temperature. Upon this " principle we might be tempted to look for it " at a ſimilar diſtance from the equator in a

 northern

" northern latitude. This has actually been

" done: Don Casimir Ortega, professor of botany at Madrid, has lately, by order of the Spanish minister for the American department, sent to the Royal Medical Society at Paris specimens of two species of *Cinchona* recently discovered in America, in the province of *Santa-Fe*, which is situated in 4½ degrees of North Lat. These specimens are well preserved, but not quite perfect, as the flowers are wanting. The leaves and fruit of one of these species exactly resemble those of the Red Bark sent by M. la Condamine, from Peru, and which are still preserved in M. de Jussieu's *Hortus Siccus*. The other specimen proves to be a White Bark, and of course a bad species. The Spanish minister accompanied these specimens with a request, that the Society would inform him what degree of attention they merited. The Society have of course given his Excellency every necessary information on this subject; and as he is now aware of the great importance of the Red Bark, there can be no doubt but proper directions will be given for its cultivation in *Santa-Fe*, not only on account of its scarcity at Loxa, but because it will be much more easily conveyed to Europe,

" as

" as a river that runs through the province of

" *Santa-Fe* empties itself into the sea near the

" harbour of Carthagena, so that we may hope

" soon to see a new source opened for this ad-

" mirable remedy."

---

At a meeting of the faculty of physic at Paris, M. Saillant read lately an account of an uncommon disease, which he considered as a species of Spina Ventosa, and to which he gave the name of Medullary Gout. The subject of it was a surgeon named Pouble, who dated the origin of his complaints from a long journey he had taken on foot about ten years before his death. From that time all his limbs were more or less painful, and by degrees became bent in different directions, and incapable of motion. His hands were constantly covered with an unctuous humour, which, as it dried, thickened into scales. His nails and the ends of his fingers were sore and painful.

His right leg and thigh were so bent, that the thigh adhered to his belly and breast, and his leg was fixed in the same manner to the thigh, so that the knee was placed almost under the axilla. The left extremity was differently twisted, as as the thigh paffed acrofs the pubis at a right angle from the left to the right, and refted on the right foot. The feet were covered with the fame greafy difcharge as the hands. The head and the different vertebræ were as ftiff and motionlefs as the other parts of the body.

The patient complained of a burning heat along his back, and a pain in his limbs, efpecially near the joints, and when expofed to the air. The urine he difcharged was high coloured, turbid, and fœtid. He was troubled with a continual itching over the whole furface of his body.

This diforder did not feem to be the true gout. There were no tophi, or appearance of anchylofis in the joints. The limbs were fixed by fimple mufcular contraction. M. Saillant compares this cafe to that of the *femme Melin* (fee our 3d vol. p. 279.)

The patient died in September 1781. On diffection all the bones were found foft and fragile. The fkin was harder, and offered more refiftance to the knife than any of the bones. In gently raifing the left thigh it broke at its upper part, and was found to confift of a foft thin bony fhell filled with marrow.

The

The articulations were almoſt entirely deſtitute of cartilage, ſo that ſome bones, as the rotula, the bones of the carpus, and ſeveral of the vertebræ were united to the neighbouring bones, or to each other by their earthy part. In ſeveral of the bones, likewiſe, the earthy part was in ſome meaſure deſtroyed. One of the thigh bones, for inſtance, the head of which was out of the cotyloid cavity, had loſt much of its earth, both at its head and about the great trochanter. The other os femoris was the only long bone, the body of which was ſenſibly vitiated.

The bones in general were ſo much lighter than uſual, that inſtead of ſinking, they ſwam in water, and required a conſiderable weight to ſink them. It had been obſerved, that the patient himſelf, when alive and put into a warm bath, floated, and required the aſſiſtance of two perſons to keep him under water. In the dead body, however, the bones were ſo porous, that they ſoon imbibed water and ſunk.

A freſh thigh bone of the ſame length as one of Pouble's weighed 13 oz. while his weighed only 4½ oz.

Eight ounces of Pouble's and the ſame weight of the bones of another ſubject were diſtilled in a retort. The latter yielded of phlegm phlegm ℥ij, gr. xx; fluid volatile alkali ʒj; gr. xij; concrete volatile alkali gr. xij; charcoal ℥v, gr. liv.—In diſtilling Pouble's bones the retort burſt, and a very penetrating volatile ſpirit inſtantly ſpread through the houſe. Notwithſtanding this accident in the proceſs it yielded of oil ℥iiß, gr. 50; concrete volatile alkali gr. xxiv; charcoal ℥iijß.

---

At a general meeting of the Society of Phyſicians of London, on Wedneſday the ſixth of Auguſt, the eulogium of their late preſident, Dr. William Hunter, was read by Dr. Simmons, who was requeſted to publiſh it.

---

The Philoſophical Society of Edinburgh has received a charter from his Majeſty, by which it is incorporated under the name of the *Royal Society of Scotland, inſtituted for the advancement of learning and uſeful knowledge.* The members are divided into two claſſes, viz. Phyſical and Literary. The objects of the firſt of theſe claſſes are to be mathematics, natural philoſophy, chemiſtry, phyſic, natural hiſtory, arts, and manufactures; factures; and of the second, antiquities, philology, and literature. The meetings of the physical class are to be held on the first Mondays of January, February, March, April, July, November, and December; and those of the literary class on the third Mondays of January, February, March, April, June, July, November, and December.——Ordinary members, that is, those who reside in or near enough to Edinburgh to attend the meetings, are to pay a guinea on their admission, and the same sum annually.—The first meeting was held on Monday the fourth of August; at this meeting the Duke of Buccleugh was elected President, and John Robinson, Esq. Professor of Natural Philosophy in the university of Edinburgh, Secretary of the Society. Two Vice-presidents, a Treasurer, and a Council, consisting of twelve members, were likewise chosen at the same time.

*New works about to be published.*——1. A Volume of Essays by the Society instituted in London for the improvement of medical knowledge.—2. Anatomical tables of the blood vessels and nerves, the brain, viscera, and parts of generation of the human body, by Mr. Andrew Bell, engraver in Edinburgh. The plates for this work are selected from the tables published by Haller, Ruysch, Du Verney, Zinn, Meckel, Walter, and others, and have been executed and arranged under the direction of Dr. Monro.—3. A treatise, in Latin, on Intermittent fevers by Dr. Strack, professor of physic at Mentz. This essay gained the prize given last year by the Academy of Dijon.—4. An account of the life and writings of the late Dr. Hunter, by Dr. Simmons.—5. Dr. Cullen is said to be preparing for the press a new and improved edition of his First lines of the Practice of physic, with the addition of a fourth volume, which is to complete the work.

