# Errata

**Published:** 1783

**Authors:** 


					p. 301, l. b, jor dc Vine read de Virieu j

				

